# Factors predicting the outcome of allergen-specific nasal provocation test in children with grass pollen allergic rhinitis

**DOI:** 10.3389/falgy.2023.1186353

**Published:** 2023-05-26

**Authors:** M. Barreto, S. Tripodi, S. Arasi, M. Landi, M. Montesano, S. Pelosi, E. Potapova, I. Sfika, V. Villella, A. Travaglini, M. A. Brighetti, P. M. Matricardi, S. Dramburg

**Affiliations:** ^1^ NESMOS Department, Faculty of Medicine and Psychology, Pediatric Unit Sant’Andrea Hospital, “Sapienza” University, Rome, Italy; ^2^ Pediatric Allergology Unit, Sandro Pertini Hospital, Rome, Italy; ^3^ Allergology Service, Policlinico Casilino, Rome, Italy; ^4^ Translational Research in Pediatric Specialities Area, Division of Allergy, Bambino Gesù Children’s Hospital, IRCCS, Rome, Italy; ^5^ Institute for Biomedical Research and Innovation, Pediatric National Healthcare System, Turin, Italy; ^6^ TPS Production., Rome, Italy; ^7^ Department of Pediatric Respiratory Medicine, Immunology and Critical Care Medicine, Charité-Universitätsmedizin Berlin, Corporate Member of Freie Universität Berlin and Humboldt-Universität zu Berlin, Berlin, Germany; ^8^ Department of Biology, Tor Vergata University, Rome, Italy

**Keywords:** seasonal allergic rhinitis, nasal provocation test (NPT), pollen allergy, component-resolved diagnostics, precision medicine, e-Diary

## Abstract

**Background:**

Nasal provocation testing (NPT) is a reference methodology to identify the culprit allergen in patients with allergic rhinitis. Selecting the right allergen for NPT is particularly difficult in poly-sensitized patients with seasonal allergic rhinitis (SAR). Predictors of NPT outcomes may facilitate the proper use of this test or even substitute it.

**Objective:**

To identify predictors of grass pollen NPT outcome from an array of clinical data, e-diary outcomes, and allergy test results in poly-sensitized pediatric patients with SAR.

**Methods:**

Poly-sensitized, SAR patients with grass pollen allergy, participating in the @IT.2020 pilot project in Rome and Pordenone (Italy), participated in a baseline (T0) visit with questionnaires, skin prick testing (SPT), and blood sampling to measure total (ImmunoCAP, TFS, Sweden) and specific IgE antibodies to grass pollen extracts and their major allergenic molecules (ESEP, Euroimmun Labordiagnostika, Germany). During the pollen season, patients filled the AllergyMonitor® e-diary app measuring their symptoms, medication intake, and allergy-related well-being via the Visual Analogue Scale (VAS). After the pollen season (T1), patients answered clinical questionnaires and underwent a nasal provocation test (NPT) with grass pollen extract.

**Results:**

We recruited 72 patients (age 14.3 ± 2.8 years, 46 males) sensitized to grass and/or other pollens, including olive (63; 87.5%) and pellitory (49; 68.1%). Patients positive to grass pollen NPT (61; 84.7%), compared to the negative ones, had worse VAS values in the e-diary, larger SPT wheal reactions, and higher IgE levels, as well as specific activity to timothy and Bermuda grass extracts, rPhl p 5 and nCyn d 1. A positive NPT to grass pollen was predicted by an index combining the specific activity of IgE towards Phl p 5 and Cyn d 1 (AUC: 0.82; *p* < 0.01; best cut-off ≥7.25%, sensitivity 70.5%, specificity: 90.9%). VAS results also predicted NPT positivity, although with less precision (AUC: 0.77, *p* < 0.01; best cut-off ≥7, sensitivity: 60.7%, specificity: 81.8%).

**Conclusions:**

An index combining the specific activity of IgE to rPhl p 5 and nCyn d 1 predicted with moderate sensitivity and high specificity the outcome of a grass pollen NPT in complex, poly-sensitized pediatric patients with seasonal allergic rhinitis. Further studies are needed to improve the index sensitivity and to assess its usefulness for NPT allergen selection or as an alternative to this demanding test procedure.

## Introduction

Allergic rhinitis (AR) is a widely prevalent condition that affects about 20% of the world population, particularly children and adolescents (1–3). Patients' discomfort is related to nasal and ocular symptoms (e.g., sneezing, rhinorrhea, nasal congestion, pruritus, tearing), systemic symptoms (e.g., fatigue, irritability, headache), and side effects of treatment (e.g., sedation by antihistamines). Severe AR symptoms lead to decreased productivity or absenteeism at school and have a negative impact on the quality of life in pediatric patients. Seasonal AR (SAR) does not only affect school performance but also participation in outdoor activities during the pollen season, and frequently coexists with asthma and other comorbidities (2, 3).

In a multicenter epidemiological study of 1,360 Italian children with seasonal allergic rhinitis, mono-sensitization was observed in only 6.2% of participants, while 84.9% were sensitized to ≥3 pollen, with timothy grass being the dominant allergenic pollen (89.6%) (4). Children with seasonal AR who cannot be sufficiently controlled with symptomatic drug treatment and preventive measures may benefit from allergen-specific immunotherapy (AIT), whose effectiveness entails the identification of the clinically relevant allergen (5). In accordance, molecular diagnostics (CRD) helps to identify, especially in poly-sensitized patients, genuine sensitization towards the major, specific molecules of pollen, thus making the AIT prescription more precise (6, 7). However, the sensitization profile expressed in the serum may diverge in some patients from the one provoking symptoms at a local level (3, 8). In these patients, testing the acute nasal response to the suspected allergen with an allergen-specific nasal provocation test (NPT) is required (3, 9). However, NPT is not easily applicable in daily clinical practice, as it is time consuming and requires preparatory actions (e.g., pausing treatments that may affect the results) and coordination (e.g., several appointments for poly-sensitized patients) (9, 10). Given these limitations, criteria have been developed to select the patients and optimal candidate allergens for NPT (9, 11). Nevertheless, diagnostic algorithms precisely predicting a positive or negative NPT outcome, aimed at reducing the need of this demanding and difficult test in daily practice, are essential. Extensive clinical research allowed predicting the outcome of oral provocation tests in food allergic patients (12–14), but similar studies, dedicated to NPT in seasonal AR, are still missing. An interesting research tool is that which assesses IgE-specific activity as the proportion of allergen-specific IgE to total IgE (15).

To fill this gap, we examined children with atopic sensitization to grass pollen, who participated in the pilot phase of the @IT.2020 study (16, 17). All children were thoroughly examined with a clinical questionnaire, pollen exposure and symptom monitoring during the pollen season, nasal cytology, skin prick test (SPT), serum-specific IgE to grass pollen extract and its molecules before undergoing an NPT to confirm or exclude a grass pollen allergy. We then extensively analyzed the data to find out which parameters or their algorithmic combination predicted a positive or negative outcome of the NPT.

## Materials and methods

### Study design

The @IT2020 pilot project is an observational clinical study on the impact of component-resolved diagnosis and digital symptom recording for the diagnosis of pollen allergy. In the context of this project, 101 participants, children aged 4–18 years suffering from seasonal allergic rhinitis, were recruited in the Sandro Pertini Hospital in Rome between November 2016 and February 2017. The detailed study protocol has been published previously (16, 17). Briefly, inclusion criteria were: (1) doctor’s diagnosis of seasonal allergic rhinitis/rhinoconjunctivitis; (2) residing within 30 km of the aerobiological station of the study center; (3) having no intention to change residence in the next 6 months; and (4) smartphone ownership (for children: parental smartphone). Exclusion criteria were: (1) previous allergen immunotherapy for any outdoor allergen; and (2) any other severe nonatopic chronic disease. Recruited patients underwent a first medical examination (T0), including skin prick testing (SPT), blood sampling, and clinical questionnaires. At the end of the visits, participants were instructed on the use of the AllergyMonitor® (AM) (TPS software production, Rome, Italy) mobile app to monitor their ocular, nasal, and lung symptoms, as well as medication intake and the impact of allergy symptoms on their well-being during an individual study period. After the monitoring period (i.e., pollination period of the suspected relevant allergen source), all participants underwent a second medical examination (T1), including a repetition of the initial clinical questionnaires focused on the past pollen season. Among the patients sensitized to grass pollen, the nasal provocation test (NPT) to grass allergens was proposed, and 82 agreed to participate. The local ethics committee approved the study design and procedures. All parents or guardians provided informed written consent to the clinical investigations.

### AllergyMonitor® App

AllergyMonitor® (AM) (TPS software production, Rome, Italy) is a CE-certified and scientifically investigated smartphone application (app) (17–22) designed for the daily reporting of allergic symptoms of the eyes, nose, and lungs. Further, the users are asked to record the impact of allergic symptoms on their daily activities and sleep, as well as their daily medication intake. To facilitate the correct recording of medication intake, the individual treatment regimen was registered individually in the app by the study doctor via back-office technology (21). While the initial set-up for each user took approximately 5 min, daily recordings could be completed within 1–2 min. AllergyMonitor output records include the Rhinoconjunctivitis Total Symptoms Score (RTSS, 0–18) (23); the Combined Symptom and Medication Score (CSMS, 0–6) (24); and the Visual Analogue Scale (VAS, 0–10) (25). Daily records of RTSS and CSMS encompass nasal symptoms (sneezing, rhinorrhea, nasal pruritus, nasal congestion), ocular symptoms (itchy eyes, watery eyes), and medication intake (antihistaminic drugs, local steroids, systemic steroids). The VAS estimates the overall AR severity by asking “How do you feel in relation to your allergic symptoms today?”.

### Grass pollen season

Season criteria of the European Academy of Allergy and Clinical Immunology (EAACI) (26) were adapted to the local setting, and for 2016 resulted in a whole grass pollen season from 13 April to 28 July, as well as a peak grass pollen season between 4 May and 28 June, as reported previously (22). Grass pollen concentrations ranged from 0 to 199 grains/m^3^ air. Based on aerobiological cut-off values for central Italy, daily pollen concentrations were also classified as low (<10/m^3^), medium (10–30/m^3^), and high (>30/m^3^) (27).

### Skin prick tests

Skin prick tests were performed using a standard panel of commercial extracts (ALK-Abelló, Milan, Italy) of outdoor and indoor aeroallergens (Alternaria, Bermuda grass, birch, cat dander, cypress, dog dander, hazel, house dust mite, mugwort, olive tree, plane tree, ragweed, Russian thistle, timothy grass, and pellitory-of-the-wall). Histamine 0.1 mg/ml and glycerol solution were used as positive and negative controls, respectively. Morrow Brown needles were used to prick the skin, and the wheal reactions were read after 15 min. A wheal equal to or greater than 3 mm after subtraction of the negative control was regarded as positive.

### IgE assays

During the T0 visit, patients underwent blood sampling. Serum total IgE was tested with ImmunoCAP-FEIA (Thermo Fisher Scientific, Phadia AB, Uppsala, Sweden), and the results were expressed in kU/L. Allergen-specific IgE antibodies were measured with a multiplex immunoblot assay (EUROLINE Southern European Profile Test EUROIMMUN Labordiagnostika AG, Lübeck, Germany) specifically designed for the *in vitro* identification of specific IgE to airborne allergen (pollen and *Alternaria alternata)* extracts in the Mediterranean area and their allergenic molecules (28). The ESEP included the following allergen extracts and native (n) and recombinant (r) molecules: birch (*Betula verrucosa*), rBet v 1, rBet v 2, rBet v 4, olive tree (*Olea europaea*), rOle e 1, cypressus (*Cupressus arizonica*), nCup a 1, Bermuda grass (*Cynodon dactylon*), rCyn d 1, timothy grass (*Phleum pratense*), rPhl p 1, rPhl p 5, rPhl p 4, rPhl p 7, rPhl p 12, mugwort (*Artemisia vulgaris*), nArt v 1, pellitory (*Parietaria judaica*), rPar j 2, Alternaria (*Alternaria alternata*), rAlt a 1 and a cross-reactive carbohydrate determinant (CCD) marker. The test is semi-quantitative, and results are expressed in kU/L. Values greater than 0.35 KU/L are considered positive. For simplicity, prefixes “n” and “r” for pollen molecules will be avoided in the text. The IgE-specific activity for grass-pollen extracts and molecules was calculated as their percentage fraction from total IgE, i.e., (specific IgE/total IgE) *100 (15). The combined IgE-specific activity was calculated as the sum of specific-IgE activities for extracts or molecules [e.g., (specific IgE to Phl p 5 + Cyn d 1/total IgE)*100].

### Nasal provocation test

During the T1 visit, patients with positive SPT for grass pollen underwent an NPT according to international guidelines (9). Baseline Total Nasal Symptom Score (TNSS), Peak Nasal Inspiratory Flow (PNIF), and subjective evaluation of symptoms such as rhinorrhea, nasal obstruction, nasal itch, ocular itch, lacrimation, were used as parameters. The evaluation system used is that proposed by the otolaryngology section of the German Society of Allergology and Clinical Immunology (11). For a detailed description of the NPT protocol, please see the supplementary material.

### Statistical analysis

Continuous variables were evaluated for normal distribution (K-S test) and reported as means ± SD or as medians and interquartile range (IQR). The Mann–Whitney *U* test was used to assess differences between two groups of continuous variables; contingency tables (*χ*^2^) with Fisher's correction were used to compare frequencies. Grass-allergen SPT variables were analyzed as the single mean-wheal size; specific IgE to extracts and molecular fractions were analyzed separately and as the sum of two or more fractions (kU/L), and as their IgE-specific activity (percentage fraction from total IgE). Individual AM daily records during the peak grass pollen season were assessed both as the maximum value and the coefficient of variation (CV = 100* SD/mean) for RTSS, CSMS, and VAS, during the days with high airborne grass-pollen concentrations (>30/m^3^). Receiver operating characteristic (ROC) curves were used to analyze the relationship between sensitivity and 1-specificity for predictors of a positive NPT; areas under curves (AUCs) were calculated. Logistic regression (enter method) was performed with the NPT outcome as a dependent variable against potentially influencing (independent) variables on the degree of allergen sensitization and symptom scores. Independent variables were selected based on significant differences between groups of NPT outcomes; the dependent variable had mutually exclusive and exhaustive categories (NPT; negative: 0, positive: 1). The Box-Tidwell test was used to estimate the linear relationship between continuous independent variables and the logit transformation of the dependent variable. Statistical software (SPSS version 27, Chicago, l) was used for calculations. Statistical significance was accepted for “P” values <.05.

## Results

### Study population

The present analysis includes 72 children and adolescents (14.3 ± 2.8 years, BMI 20.7 ± 3.8 kg/m^2^, BMI percentile 57.1 ± 30.3, M/F: 46/26) fulfilling the inclusion criteria for the @IT.2020 pilot study and with a complete dataset of assessments including the NPT. The male gender was slightly more frequent (63.4%), and the population was characterized by a predominantly persistent classification of AR symptoms as assessed by a retrospective questionnaire during T0 according to the Allergic Rhinitis and Its Impact on Asthma (ARIA) criteria; persistent symptoms were reported as moderate to severe in 40.3% (29/72) of patients in this session. The rate of patients with moderate-severe persistent symptoms increased to 75% (54/72, *p* < 0.01) at the final study visit (T1) when being asked the same questions concerning the past grass pollen season. Frequent allergic comorbidities were oral allergy syndrome, asthma, and atopic dermatitis. All participants were sensitized to grass pollen, with 98.6% having a positive SPT to timothy grass and 90.3% reacting to Bermuda grass; they also were frequently sensitized to other pollens, mostly olive, cypress, and wall pellitory (Table 1).

**Table 1:** Characteristics of the study population at T0.

Allergic rhinitis	
Age at onset, median (IQR)	5.5 (4.0–7.7)
**Aria class, *n* (%)**	
mild intermittent	14 (19.4)
moderate-severe intermittent	9 (12.5)
mild persistent	20 (27.8)
moderate-severe persistent	29 (40.3)
**Lifetime comorbidities, n (%)**	
Asthma	21 (29.2)
Oral allergic syndrome	23 (31.9)
Atopic dermatitis	18 (25.0)
Urticaria-angioedema	13 (18.1)
Gastrointestinal disorders	4 (5.6)
Anaphylaxis episodes	6 (8.3)
Other allergies	3 (4.2)
**Whole allergen sensitization, median (IQR)**	
IgE tot ImmunoCAP, kU/L, median (IQR)	421 (199–737)
SPT wheals ≥3 mm median (IQR)	10 (7–12)
**Skin prick test results to aeroallergens (wheals ≥3 mm)**	
*Outdoor, n (%)*	
Timothy grass	71 (98.6)
Bermuda grass	65 (90.3)
Olive tree	63 (87.5)
Cypress	59 (81.9)
Birch	30 (41.7)
Hazel	29 (40.3)
Wall pellitory	49 (68.1)
Plane tree	39 (54.2)
Alternaria	34 (47.2)
Russian thistle	30 (41.7)
Mugwort	20 (27.8)
Ragweed	25 (34.7)
*Indoor, n (%)*	
House dust mite	49 (68.1)
Cat dander	51 (70.8)

Variables are medians (interquartile range), or numbers (percentages).

### NPT outcomes

Of the 72 patients, 61 (84.7%) had a positive NPT to grass pollen extract. Patients' anthropometric data, comorbidities, and therapy for AR did not differ by NPT outcomes. All subjects were poly-sensitized to aeroallergens; the median (IQR) number of SPT wheal reactions ≥3 mm was similar in patients who had a positive or a negative NPT [10 (7–12) vs. 9 (8–10)], *p* = 0.937 by Mann–Whitney *U* test. Out of the 11 patients with a negative NPT, nine (81.8%) and three (27.3%) had no IgE to Phl p 5 and to Phl p 1, respectively. Four and two of these patients were highly sensitized, both *in vivo* (SPT) and *in vitro* (IgE), to pellitory and to olive, respectively. Only two of the 11 patients (18.2%) were highly sensitized to grass pollen, having IgE to Phl p 5, so that a negative NPT could not be easily explained.

### IgE sensitization vs. NPT outcomes

Among the specific IgE to timothy grass molecules, only IgE to Phl p 5 predicted NPT outcomes. IgE levels to Phl p 5 > 0.35 KU/L were found in 39/61 (64.0%) of NPT-positive subjects vs. 2/11 (18.2%) of NPT-negative subjects (*p* = 0.007 by *χ*^2^ Fisher's corrected). IgE to Phl p 1 and IgE to Phl p 4 were observed in almost all the patients despite their NPT outcome; conversely, IgE to Phl p 7 and Phl p 12 were infrequently observed in both patient groups. Overall, patients with a positive NPT were more intensively sensitized to grass pollen than patients yielding negative NPT, as they had larger SPT wheal reactions to timothy grass and Bermuda grass, and higher specific IgE levels to timothy grass extract, to Phl p 5, and to Cyn d 1 (Table 2). This difference was even stronger when the specific activity of the above listed IgE antibodies was considered (Table 3).

**Table 2:** Patient characteristics by outcomes of the nasal provocation test (NPT).

Variables	Negative (*n* = 11)	Positive (*n* = 61)	*p*-values
Males/females	9/2	37/24	0.307
Age, year	14.0 ± 2.1	14.4 ± 2.9	0.850
BMI, kg/m^2^	20.3 ± 3.4	20.8 ± 3.9	0.888
BMI percentile	54.2 ± 31.1	57.6 ± 30.4	0.650
**Aria class at T1, nYes/nNo (%Yes)**			
mild intermittent	1/10 (9.1)	4/57 (6.6)	
moderate-severe intermittent	1/10 (9.1)	1/60 (1.6)	
mild persistent	2/9 (18.2)	9/52 (14.8)	
moderate-severe persistent	7/4 (63.6)	47/14 (77.0)	0.523
**Comorbidities, past 12 months nYes/nNo (%Yes)**			
Asthma	4/7 (36.4)	17/44 (27.9)	0.720
Oral allergic syndrome	1/10 (9.1)	15/46 (24.6)	0.436
Atopic dermatitis	0/11 (0.0)	7/54 (11.5)	0.585
Urticaria-angioedema	2/9 (18.2)	1/60 (1.6)	0.059
Other allergies	1/10 (9.1)	1/60 (1.6)	0.284
**Grass allergen sensitization, median (IQR)**			
Timothy grass, SPT mm	6.0 (5.0–7.0)	8.0 (6.8–10.8)	0.003
Bermuda grass, SPT mm	4.5 (3.0–5.0)	6.0 (4.5–7.8)	0.003
Total IgE, ImmunoCAP, kU/L	632.6 (138.2–760.6)	402.8 (207.1–729.1)	0.568
Specific IgE Bermuda grass, kU/L	1.7 (0.4–36.0)	29.0 (2.3–59.5)	0.095
Specific IgE timothy grass, kU/L	65.0 (3.4–97.0)	95.0 (72.0–100.0)	0.029
**Specific IgE grass-pollen molecules, kU/L**			
Phl p 1	72.0 (20.0–96.0)	91.0 (78.0–99.0)	0.097
Phl p 4	56.0 (2.7–72.0)	57.0 (31.0–71.0)	0.725
Phl p 5	0.10 (0.10–0.10)	17.4 (0.10–55.0)	0.008
Phl p 7	0.10 (0.10–0.10)	0.10 (0.10–0.10)	0.545
Phl p 12	0.10 (0.10–0.10)	0.10 (0.10–0.10)	0.524
Cyn d 1	1.7 (0.1–38.0)	37.0 (1.2–71.0)	0.019
**Therapy for AR, *n* (%)**			
Oral antihistamines	7 (63.6)	45 (73.8)	0.485
Topic antihistamines	4 (36.4)	10 (16.4)	0.207
Oral corticosteroids	0 (0.0)	5 (8.2)	0.425
Topic corticosteroids	9 (81.8)	47 (77.0)	0.539
**AR VAS in the past grass pollen season**			
VAS drug efficacy	7.0 (2.0–8.0)	8.0 (6.5–8.5)	0.269
VAS severity	5.0 (5.0–6.0)	7.0 (6.0–8.0)	0.004
**AllergyMonitor values, median (IQR)**			
RTSS maximum^a^	7.0 (2.0–10.0)	9.0 (4.0–12.0)	0.275
RTSS CV, %	120.0 (66.8–158.5)	93.6 (69.9–145.6)	0.536
CSMS maximum^a^	3.0 (2.0–3.0)	3.0 (2.0–3.7)	0.341
CSMS CV, %	62.2 (24.3–113.3)	65.2 (36.5–98.4)	0.988
VAS maximum^a^	4.0 (0.0–6.0)	6.0 (4.0–7.0)	0.017
VAS CV, %	54.3 (0.0–97.9)	90.7 (60.7–143.2)	0.019

^a^ Maximum values recorded by patients during the days with high airborne grass-pollen concentrations (>30/m^3^). CV, coefficient of variation (100*SD/mean) for individual scores during the days with high environmental grass-pollen counts. RTSS, rhinoconjunctivitis total symptom score; CSMS, combined symptom and medication score; VAS, visual analogue scale (0–10). Variables are medians (interquartile range) or numbers (percentages). *p*-values were assessed by *χ*^2^ (Fisher) or Mann–Whitney tests.

**Table 3:** Values of IgE-specific activity to grass-pollen extracts and molecules by outcomes of the nasal provocation test (NPT) with the undiluted grass pollen extract.

Variables	Negative (*n* = 11)	Positive (*n* = 61)	*p*-values
**IgE-specific activity to extracts, %**			
Timothy grass	9.10 (4.28–15.31)	20.08 (9.86–31.29)	0.004
Bermuda grass	1.57 (0.14–5.70)	5.64 (0.57–10.30)	0.024
Timothy + Bermuda	9.24 (4.36–19.06)	27.73 (14.25–42.26)	0.001
**IgE-specific activity to molecules, %**			
Phl p 1	10.8 (5.0–15.2)	20.9 (9.8–37.2)	0.018
Phl p 5	0.04 (0.01–0.22)	4.04 (0.04–11.93)	0.010
Cyn d 1	0.76 (0.07–6.01)	6.61 (0.53–13.26)	0.007
Phl p 5 + Cyn d 1	0.98 (0.15–7.24)	14.53 (3.50–24.21)	<0.001

The IgE-specific activity for extracts (timothy grass, Bermuda grass), grass-pollen molecules (Phl p 5, Cyn d 1), and their combined activity was calculated as percentages of total IgE: [(specific IgE/total IgE)*100].

Values are medians (interquartile range). The Mann–Whitney test was used for comparisons.

### AR symptoms vs. NPT outcomes

Patients with a positive NPT also perceived a higher impact of their AR symptoms on disease severity than their counterparts with a negative NPT. This difference was coherently observed not only retrospectively at T1, by the VAS for AR severity in the past grass pollen season, but also prospectively by the VAS measured during the high grass pollen season (Table 2). In contrast, RTSS and CSMS values did not differ among patients with different NPT outcomes.

### Individual predictors of the NPT outcome

**Figure 1:**
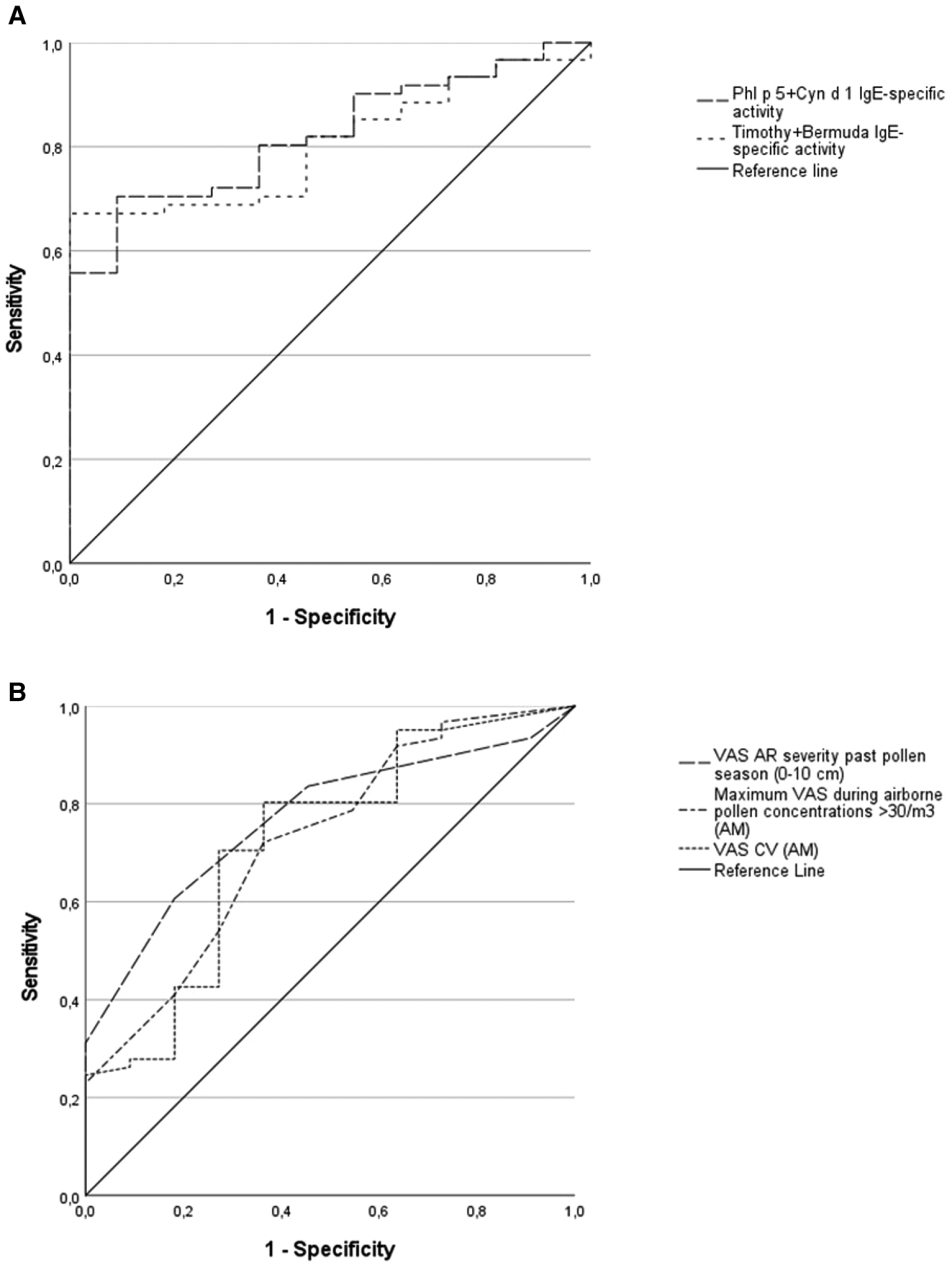
Receiver operating characteristic (ROC) curves for allergen sensitization and AR visual analogue scales (VAS) as predictors of the nasal provocation test (NPT) outcome. 1-A: combined IgE-specific activity to timothy plus Bermuda grass extracts, area under curve (AUC) = 0.81; combined IgE-specific activity to Phl p 5 plus Cyn d 1, AUC = 0.82; *p* < 0.01 for both. 1-B: VAS for AR severity in the past grass pollen season, AUC = 0.77, *p* < 0.01; maximum VAS recorded (AllergyMonitor, AM) during the days with high airborne grass-pollen counts (>30/m^3^), AUC = 0.73, *p* < 0.05; VAS coefficient of variance (VAS CV, AM) during the days with high airborne grass-pollen counts, AUC = 0.72, *p* < 0.05.

High diagnostic values on predicting a positive NPT were found for the combined IgE-specific activity to either timothy plus Bermuda grass extracts, or to Phl p 5 plus Cyn d 1 (Figure 1A). Less accurate predictors of a positive NPT were the SPT wheal size or the specific IgE level (or the IgE-specific activity), for a single grass-allergen extract or molecule (Table 4). ROC curves for the AR severity score (VAS) in the past grass pollen season fitted better than both the e-diary AR VAS records (CV and maximum VAS) during airborne grass-pollen concentrations >30/m^3^ (Figure 1B).

**Table 4:** ROC analysis for grass-pollen sensitization and symptom scores on AR severity by outcomes of the nasal provocation test (NPT).

	AUC	OQM	Interruption	Sensitivity	Specificity	PPV	NPV
**SPT, mm**							
Timothy grass	0.78	0.67	7.75	57.4	90.9	97.2	27.8
Bermuda grass	0.78	0.68	5.75	57.4	100.0	97.2	29.7
**Specific IgE extracts, kU/L**							
Timothy grass	0.70	0.53	65.5	85.2	54.5	91.2	39.9
Timothy grass activity, %	0.78	0.67	16.07	65.6	90.9	97.6	32.3
Bermuda grass	0.66	0.51	1.90	77.0	54.5	90.4	31.5
Bermuda grass activity, %	0.72	0.59	6.41	49.2	100.0	100.0	26.2
Timothy + Bermuda	0.71	0.54	66.55	86.9	54.5	91.4	42.9
Timothy + Bermuda activity,%	0.81	0.70	21.7	67.2	100.0	100.0	35.5
**Specific IgE molecules, kU/L**							
Phl p 5	0.74	0.60	1.10	63.9	81.8	95.1	29.0
Phl p 5 activity, %	0.74	0.62	0.98	64.0	90.9	97.5	31.3
Cyn d 1	0.72	0.58	56.50	39.3	100.0	100.0	22.9
Cyn d 1 activity, %	0.76	0.62	7.27	47.5	100.0	100.0	25.6
Phl p 5 + Cyn d 1	0.76	0.62	55.75	52.5	90.9	97.0	25.6
Phl p 5 + Cyn d 1 activity, %	0.82	0.72	7.25	70.5	90.9	97.7	35.7
**AR severity scales, 0–10**							
Maximum VAS (AM)	0.73	0.57	4.50	72.1	63.6	91.2	29.2
VAS CV (AM), %	0.72	0.55	55.15	80.3	63.6	92.4	36.8
VAS past grass pollen season	0.77	0.64	6.50	60.7	81.8	94.9	27.3

AUC, area under curve; OQM, overall quality of the model. Interruption: best cutoff value. PPV, positive predictive value; NPV, negative predictive value; SPT, skin prick test.

The IgE-specific activity for extracts (timothy grass, Bermuda grass), grass-pollen molecules (Phl p 5, Cyn d 1), and their combined activity was calculated as percentages of total IgE: [(specific IgE/total IgE)*100].

VAS, visual analogue scale (0–10). AM, allergyMonitor; Maximum VAS, maximum records during the days with high airborne grass-pollen counts (>30/m^3^). CV, coefficient of variation (100*SD/mean) for individual scores during the days with high environmental grass-pollen counts.

### Algorithm best predicting NPT outcome

**Figure 2:**
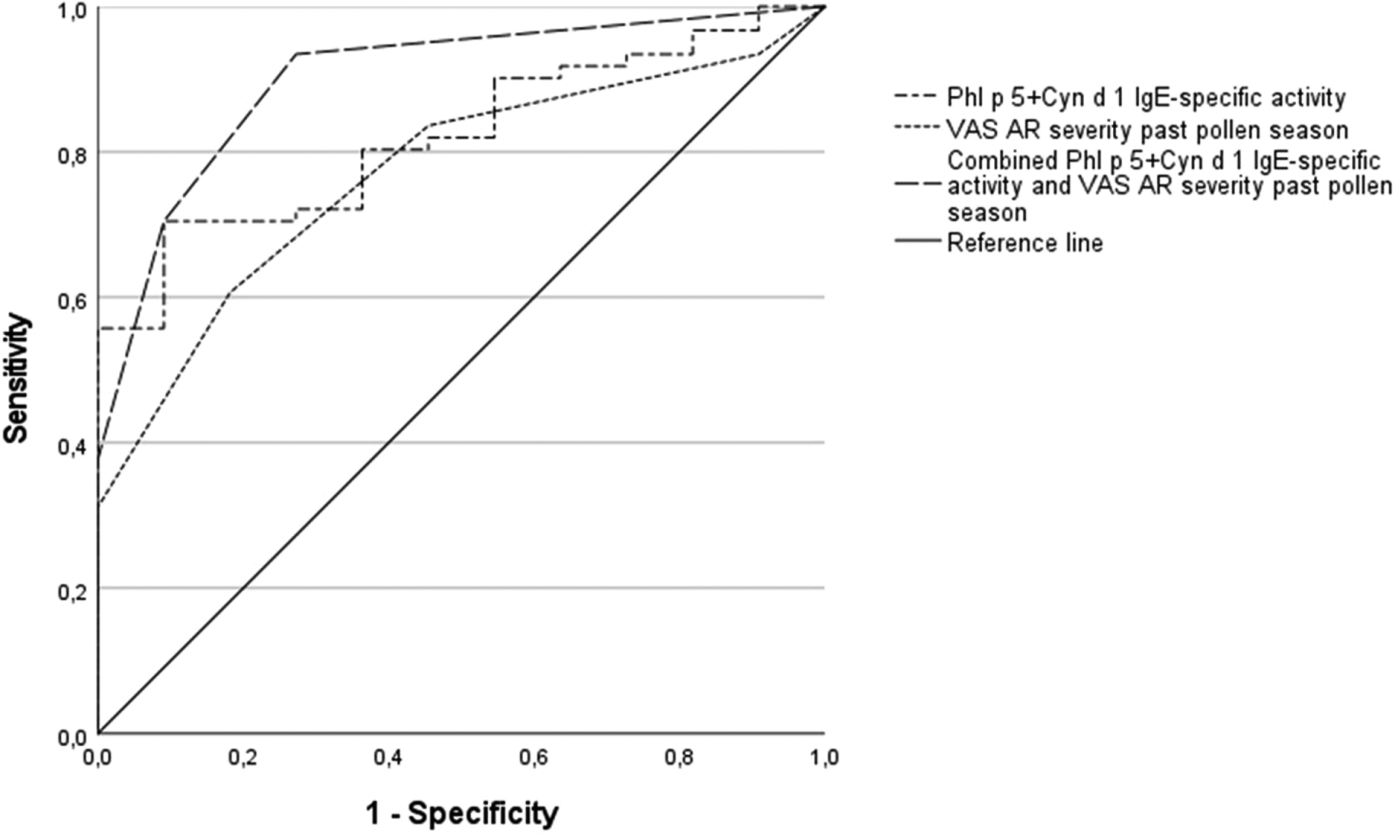
Receiver operating characteristic (ROC) curves for sensitization to combined grass molecules and AR visual analogue scales (VAS) as predictors of the nasal provocation test (NPT) outcome. Combined IgE-specific activity to Phl p 5 plus Cyn d 1, AUC = 0.82, *p* < 0.01; VAS for AR severity in the past grass pollen season, AUC = 0.77, *p* < 0.01; diagnostic algorithm using cut-off values for the combined IgE-specific activity (Phl p 5 plus Cyn d 1 ≥7.25%) or VAS scores for AR severity in the past pollen season ≥7, AUC = 0.90, *p* < 0.001.

We tested the hypothesis that the combination of multiple predictors in a diagnostic algorithm may improve our predictive capacity. When NPT outcomes were tested with two potential explanatory variables (degree of allergen sensitization and symptom scores), the best logistic model included the combined IgE-specific activity for Phl p 5 plus Cyn d 1 and the VAS for AR severity in the past grass pollen season (Table 5). The regression model was statistically significant (*χ*^2^ = 22.85, *p* = 0.000), explained 47% of the variance (Nagelkerke R square), and correctly classified 86.1% of cases. A raise in the combined IgE-specific activity for Phl p 5 plus Cyn d 1 and the VAS for AR severity in the past grass pollen season was associated with an increased likelihood of having a positive NPT outcome (Odds ratio 1.2 and 2.4, respectively) (Table 5). Based upon optimal cut-off values for ROC curves in Table 4, percentages of IgE-specific activity for Phl p 5 plus Cyn d 1 ≥7.25% or VAS scores for AR severity in the past pollen season ≥7, predicted the NPT outcome with a 93% sensitivity, 73% specificity, 95% PPV, and 67% NPV in our patients (Figure 2).

**Table 5:** Predictors of a positive nasal provocation test (NPT) with the undiluted grass pollen extract. Variables in the equation.

							95% C.I. for Exp (B)	
	B	SE	Wald	g.l.	sign	Exp (B)	lower	upper
AR VAS past pollen season	0.886	0.334	7.029	1	0.008	2.425	1.260	4.667
Phl p 5 + Cyn d 1 activity	0.209	0.085	6.078	1	0.014	1.232	1.044	1.454
Constant	−5.300	2.223	5.684	1	0.017	0.005		

AR VAS: self-reported AR severity scale (0–10) in the past pollen season.

Phl p 5 + Cyn d 1 activity: combined-IgE specific activity of both grass-pollen molecules.

i.e., (Phl p 5 + Cyn d 1/total IgE)*100.

Exp (B): odds ratio with 95% confidence intervals.

## Discussion

In our study group of pediatric patients allergic to grasses, both the degree of specific allergen sensitization and self-reports of AR severity predicted the NPT outcome well; we also found that the combination of IgE-specific activity of Phl p 5 plus Cyn d 1 together with the VAS value of AR severity in the past grass pollen season predicted with a high sensitivity and moderate specificity the outcome of an NPT with grass pollen extract. In contrast, anthropometric characteristics, ARIA criteria, comorbidities, and medication use for AR did not predict the NPT outcome. To our knowledge, this is the first study investigating complex diagnostic algorithms, based on multiple variables, which may reduce the need to perform NPT in patients with seasonal allergic rhinitis due to grass pollen allergy.

Despite involving risk, the need for standardization, time consumption, and costs, NPT remains the reference test for assessing the clinical relevance of an allergen (9). A systematic review and meta-analysis including seven studies (430 patients) on several airborne allergens reported a pooled estimate for sensitivity and specificity for SPT results (“positive” or “negative”) of 85% and 77% respectively on predicting the NPT outcome (29); three of these studies testing timothy grass reported sensitivity between 68% and 97% and specificity between 70% and 86% (30–32). To note, different cut-off values for defining a “positive” SPT were used (29). The SPT-allergen wheal size and/or specific IgE levels have been found predictive of NPT results for several allergens such as *Dermatophagoides pteronyssinus (D. pt.)* (33, 34), cat (35, 36), and *Salsola kali* (37); in contrast, the SPT wheal size was found unrelated to timothy grass-titrated NPT outcomes in a study by Huss-Marp et al. (38). Notwithstanding the absent dose-response relationship, the authors could establish that specific IgE levels for timothy extract at the cut-off 0.35 kUA/L predicted dichotomized challenge outcomes (any reaction regardless of concentration vs. no reaction), with a sensitivity of 99% and specificity of 84% (38).

Sensitization to multiple molecular allergen components has been found predictive of the acute response to the specific mucosal challenge. Darsow et al., in 101 adult patients with timothy grass allergy, analyzed IgE against eight molecules (Phl ps: 1, 2, 4, 5b, 6, 7, 11, and 12). Increased numbers of sensitizations to these molecules (cut-off 0.35 kUA/L) predicted NPT and conjunctival provocation test (CPT) outcomes (39). We found that only IgE to Phl p 5 was significantly more frequent (64.0%) in NPT-positive subjects than in NPT-negative subjects (18.2%); other serological parameters, such as IgE to Phl p 1 and Phl p 4 (both very frequent) or IgE to Phl p 7 and Phl p 12 (both relatively infrequent) did not add any further power to our prediction capacity of NPT outcomes. Phl p 5, a prototypic member for the group 5 allergen molecules of grass pollen, is an important molecule, whose potent allergenicity is probably due to its multiple, independent IgE epitopes (40). Grass pollen allergic patients with IgE to Phl p 5 were shown to have a higher risk of developing asthma, with an increasing prevalence of sensitization to this molecule towards adulthood (41, 42). Consistent with these previous observations, our study adds a new, hitherto unrecognized property of sensitization to Phl p 5, which confirms the diagnostic relevance of *in vitro* molecular testing for a complete phenotype analysis of the patients with grass pollen allergy (43), as well as standardization of grass pollen AIT preparation guided by their content of Phl p 5 (44).

Estimates of the IgE-specific activity as the proportion of allergen-specific IgE to total IgE have been suggested as more appropriate than allergen-specific IgE levels for calculating the degree of allergen sensitization and, ultimately, its impact on clinical symptoms (28, 45). We calculated the IgE-specific activity for main grass-pollen extracts but also for grass-pollen molecules, an approach that (to our knowledge) has not been used before to assess NPT predictors in poly-sensitized patients. Our results confirm the relevance of IgE-specific activity as a novel parameter that may be introduced in routine clinical practice (12, 28, 46, 47). However, it would be important to test whether such a valuable performance can be replicated not only in other populations of grass pollen allergic patients, but also among patients mainly sensitized to other pollen (e.g., birch, pellitory, olive).

Certainly, the severity of symptoms upon allergen exposure does not depend solely on IgE-specific activity, but also on other immunological parameters, host factors, and environmental factors, particularly regarding exposure to other, co-seasonal allergenic pollen (45). Hence, we temporally restricted our analysis on the season segment characterized by high airborne grass-pollen concentrations (peak pollen season) to minimize the confounding effect of overlapping airborne allergens (e.g., olive, pellitory), to which many of our patients were also co-sensitized. The relevance of peak pollen seasons and so-called “high days” has been already highlighted by previous studies (48, 49) and included in consensus statements of the EAACI (25). Our study offers an additional reason to keep in consideration those guidelines and focus on high pollination periods, especially when examining patients with poly-sensitization to co-seasonal pollen.

Contrasting results have been reported on the relationship between clinical history (self-reported AR symptoms) and NPT outcomes. AR severity, as assessed by the Rhinoconjunctivitis Quality of Life Questionnaire (RQLQ) score, increased with the dose of titrated Dpt-NPT (50), but another index of AR severity, the Total Nasal Symptom Score (TNSS) did not relate with the allergen concentration to elicit a positive *D. pt.*-NPT (34). Similarly, the VAS referring to the most recent pollen season was found unrelated to results from titrated-NPT for grasses (51). In contrast, we found that both retrospective self-reported nasal symptoms/VAS and prospectively daily reported VAS values predicted NPT outcomes. Not surprisingly, we also found in this population sample a close relationship between retrospective and prospective assessments for symptom severity of seasonal AR (52). The assessment of exposure-related symptoms may also serve as a prognostic marker for NPT-outcomes in patients suffering from local allergic rhinitis. However, future studies will be needed to carefully evaluate this potential.

A relevant advancement in our study, compared to previous ones, is that the combination of biological (atopic sensitization to grass pollen) and clinical (retrospective and prospective disease severity scores) methods is an essential strategy to win a better prediction power. This approach replicates what has been already repeatedly demonstrated in patients with food allergies, where diagnostic algorithms predicting the outcome of oral provocation tests with the culprit food are more efficient if they combine both biological and clinical information (53, 54, 14).

We must acknowledge some limitations of our study protocol. First, we performed the NPT with undiluted allergen extract rather than allergen titration; hence we could not establish a sensitivity threshold to allergens and quantitative response through a dose-finding process (9, 55). However, our procedure is in line with the recommendation for qualitative outcomes (positive vs. negative) expressed in recently published international guidelines (8). Second, we examined only 11 NPT-negative patients. However, we selected patients for a “real-life” clinical study in pediatric patients with seasonal AR symptoms and allergen poly-sensitization including grass pollen, whose symptoms justified the NPT procedure. Moreover, the observed associations and predictions were all examined for their statistical significance, so that this limitation may apply, if at all, only to the negative outcomes, not to the positive ones. The small sample size of NPT-negative patients may have affected the statistical power and generalizability of the findings as observed for the low negative predictive value and specificity of our predictive algorithm.

In conclusion, the combined IgE-specific activity for main allergen molecules of timothy and Bermuda grass together with the retrospective and prospective VAS score for AR severity helps to predict the outcome of allergen-specific NPT for grass pollen extracts in pediatric patients of a Mediterranean country sensitized to multiple pollen with overlapping seasonality. Measuring these parameters can help appropriate NPT prescription and even reduce the need for time-consuming NPT in such patients.

## Data Availability

The data that support the findings of this study are available from the corresponding author upon reasonable request.
