# Supplementary material for: Factors predicting the outcome of allergen-specific nasal provocation test in children with grass pollen allergic rhinitis
Source: Front Allergy. 2023 May 26;4:1186353. doi: 10.3389/falgy.2023.1186353 (PMC10250668; doi:10.3389/falgy.2023.1186353)
Supplement: Supplementary file 1 [file Datasheet1.pdf]

Factors predicting the outcome of allergen specific nasal provocation test in children with grass pollen allergic rhinitis.

Barreto M[1], Tripodi S[2,3], Arasi S[4], Landi M[5], Montesano M[1], Pelosi S[6], Potapova E[7], Sfika I[2], Villella V[2], Travaglini A[8], Brighetti MA[8], Matricardi PM[7], Dramburg S[7]*

From:

[1] NESMOS Department, Faculty of Medicine and Psychology, Pediatric Unit Sant'Andrea Hospital, "Sapienza" University, Rome , Italy.
[2] Pediatric Allergology Unit, Sandro Pertini Hospital, Rome, Italy
[3] Allergology Service, Policlinico Casilino, Rome, Italy
[4] Translational Research in Pediatric Specialities Area, Division of Allergy, Bambino Gesù Children's Hospital, IRCCS, Piazza Sant'Onofrio, 4, 00165 Rome, Italy
[5] Pediatric National Healthcare System, Turin, Italy.
[6] TPS Production., Rome, Italy.
[7] Department of Pediatric Respiratory Medicine, Immunology and Critical Care Medicine, Charité-Universitätsmedizin Berlin, Corporate Member of Freie Universität Berlin and Humboldt-Universität zu Berlin, 13353 Berlin, Germany
[8] Department of Biology, Tor Vergata University, Rome, Italy.

Words: 559

Corresponding author:
Stephanie Dramburg
Department Pediatric Respiratory Care, Immunology and Critical Care Medicine
Charité Universitätsmedizin Berlin
Augustenburgerplatz, 1
13353 Berlin, Germany
+49 30 450 566 406
+49 30 450 566 931
stephanie.dramburg@charite.de

Methods:

The TNSS (0-7) is given by the sum of the scores assigned to nasal discharge (range 0-2), sneezing in the previous 15 minutes (range 0-3), and extra nasal symptoms (tearing, itchy eyes/throat, conjunctivitis/chemosis, urticaria, cough/dyspnea) range 0-2 **(1)**. The Peak Nasal Inspiratory Flow (PNIF) was evaluated by means of the Peak Nasal Inspiratory Flowmeter (GM-Instruments, Irvine, United Kingdom). The instrument mask was positioned hermetically and adhered to the patient's face to cover his nose and mouth. The patient was asked to exhale and then inhale vigorously through the nose while keeping the mouth closed. The best of 3 surveys with <10% variation was considered **(2)**. In addition, patients were asked to complete a Visual Analogue Scale (VAS) from 0 to 10 for symptoms such as rhinorrhea, nasal obstruction, nasal itch, ocular itch, and tearing. For the execution of NPT were required: a) consent of the patient; b) absence of anterior rhinoscopy of pathological findings such as severe turbinate hypertrophy, crusts, abundant nasal secretions, severe deviation of the nasal septum, nasal polyposis; c) drug abstention (7 days for local antihistamine and steroid therapy, 21 days for systemic steroids, 6 days for sodium cromoglycate, 7 days for non-steroidal anti-inflammatory drugs, 28 days for immunosuppressive drugs); d) absence of contraindications (basal TNSS> 2, acute rhinitis or sinusitis, exacerbation of allergic diseases such as rhinitis, food allergy, allergy to Hymenoptera venom, allergy to drugs or urticaria, previous anaphylactic reaction to the allergen, poorly controlled asthma, COPD, heart disease, pregnancy). Once all the previous conditions had been verified, the negative control (galenic solution of glycerol at 63%, at room temperature) was administered through a spray dispenser 1 puff per nostril equal to 0.09 ml/puff. The dispenser applicator was inserted into the nasal vestibule facing upwards and laterally towards the medial canthus of the ipsilateral eye, to deposit the solution on the mucosa of the middle and lower nasal turbinates. While administering the spray, patients were asked to bow their heads forward and vocalize to minimize the risk of lower airway inhalation. In the following 10 minutes, the signs and symptoms evoked by the stimulus were recorded and at the end of the observation the TNSS, PNIF, and VAS were re-evaluated and, in case of negative, 1 puff per nostril of allergen extract was administered undiluted and at room temperature, utilizing Graminacee blend, 300 SRU/ml (ALK Abellò - Milan, Italy) which has an allergen concentration of Phl p 5 equals to 26 mcg/ml. While administering the spray, patients were asked to vocalize to minimize the risk of inhalation into the lower airways. In the following 15 minutes, the signs and symptoms evoked by the stimulus were recorded and

at the end of the observation the TNSS, PNIF, and VAS were re-evaluated. The nasal provocation test was considered positive if it had occurred one or more of the following conditions: a) decrease in PNIF greater than 40% compared to baseline; b) TNSS equal to or greater than 3; c) TNSS equal to 2 together with a reduction in PNIF greater than 20% from baseline. In the case of a positive result to control solution, the test was stopped. At the end of the NPT, antihistamine therapy (Cetirizine) and topical (nasal) steroid therapy were administered. In case of the onset of intense itching in the throat, dysphonia, or dysphagia, Budesonide 2 mg per aerosol was dispensed.

References:

1. Riechelmann H, Bachert C, Goldschmidt O, Hauswald B, Klimek L, et al. German Society for Allergology and Clinical Immunology (ENT Section); Working Team for Clinical Immunology. Application of the nasal provocation test on diseases of the upper airways. Position paper of the German Society for Allergology and Clinical Immunology (ENT Section) in cooperation with the Working Team for Clinical Immunology. Laryngorhinootologie. 2003 Mar;82(3):183-8. doi: 10.1055/s-2003-38411. PMID: 12673517.

2. Nathan RA, Eccles R, Howarth PH, Steinsvåg SK, Togias A. Objective monitoring of nasal patency and nasal physiology in rhinitis. J Allergy Clin Immunol. 2005;115(3 Suppl 1):S442-59. doi: 10.1016/j.jaci.2004.12.015. PMID: 15746882; PMCID: PMC7112320.
